# Distribution of vascular epiphytes along a tropical elevational gradient: disentangling abiotic and biotic determinants

**DOI:** 10.1038/srep19706

**Published:** 2016-01-22

**Authors:** Yi Ding, Guangfu Liu, Runguo Zang, Jian Zhang, Xinghui Lu, Jihong Huang

**Affiliations:** 1 Institute of Forest Ecology, Environment and Protection, Chinese Academy of Forestry, Beijing, 100091 China; 2 Institute of Resource Insects, Chinese Academy of Forestry, Kunming, 650224, China; 3 State Key Laboratory of Forest and Soil Ecology, Institute of Applied Ecology, Chinese Academy of Sciences, Shenyang 110164, China; 4 Section for Ecoinformatics and Biodiversity, Department of Bioscience, Aarhus University, Aarhus C, DK-8000, Denmark

## Abstract

Epiphytic vascular plants are common species in humid tropical forests. Epiphytes are influenced by abiotic and biotic variables, but little is known about the relative importance of direct and indirect effects on epiphyte distribution. We surveyed 70 transects (10 m × 50 m) along an elevation gradient (180 m–1521 m) and sampled all vascular epiphytes and trees in a typical tropical forest on Hainan Island, south China. The direct and indirect effects of abiotic factors (climatic and edaphic) and tree community characteristics on epiphytes species diversity were examined. The abundance and richness of vascular epiphytes generally showed a unimodal curve with elevation and reached maximum value at ca. 1300 m. The species composition in transects from high elevation (above 1200 m) showed a more similar assemblage. Climate explained the most variation in epiphytes species diversity followed by tree community characteristics and soil features. Overall, climate (relative humidity) and tree community characteristics (tree size represented by basal area) had the strongest direct effects on epiphyte diversity while soil variables (soil water content and available phosphorus) mainly had indirect effects. Our study suggests that air humidity is the most important abiotic while stand basal area is the most important biotic determinants of epiphyte diversity along the tropical elevational gradient.

Understanding the mechanisms of species distributions at different spatial scales remains a central question of community ecology and biogeography^1^. In natural ecosystems, species diversity is influenced both directly and indirectly by a set of abiotic and biotic variables^2^,^3^. These factors play different roles in determining species distribution and abundance. Distinguishing the relative importance of direct and indirect factors would greatly improve our understanding of species distribution mechanisms as well as help promote biodiversity conservation in complex natural ecosystems^2^.

Epiphytes are a well-known feature in tropical rain forests^4^,^5^ and they contribute to the local and regional floristic diversity^4^. Many biotic and abiotic factors determine the abundance and diversity of epiphytes, such as climate, water availability, edaphic factors, host tree size, species identity, bark features, and tree architecture^6^,^7^,^8^,^9^. Indirect effects such as enhanced nutrients from decomposing leaves influences epiphytes greatly^7^. Terrestrial soil nutrients and water content also indirectly influence epiphyte establishment by directly affecting the structure and community of host trees^8^. The relative importance of direct abiotic factors influencing epiphyte communities is well studied^9^,^10^,^11^,^12^,^13^. However, little is known about the relative importance of indirect effects of abiotic and biotic factors on epiphyte distribution.

Most studies addressing epiphyte diversity focus on relationships with host trees^8^,^14^ or the distribution in a single vegetation type^6^,^15^,^16^. However, diversity patterns and mechanisms determining epiphyte distributions across environmental gradients is still under explored^10^,^13^,^14^,^17^. On a large geographic scale, climate, disturbance, and evolutionary history all influence species diversity^13^. The relative importance of different abiotic and biotic variables can change greatly with variations in the macro-environment. For example, examining species distribution along an elevational gradient in a single montane area might exclude the influence of geological history, which could strongly affect the species diversity. Studying distribution patterns of epiphytes along an elevational gradient will improve our understanding of how range-restricted epiphyte species^17^,^18^ are distributed in natural ecosystems and the mechanisms that underlie spatial patterns in species diversity.

In the following study we use vascular epiphyte data from a field investigation along a tropical elevational gradient on Hainan Island, south China to examine the limiting factors on epiphyte distributions. We asked the following two questions: (1) How does abundance, richness and composition of epiphytes change along elevational gradients? (2) What are the key abiotic and biotic factors related to epiphyte diversity? Most vascular epiphytes root in the crowns of host trees rather than on the ground^4^, so we hypothesized that climate variables (especially relative humidity of the air) would directly determine epiphyte diversity along an elevational gradient. The host trees provide habitats for establishment and growth of vascular epiphytes^14^, meanwhile host trees are directly influenced by soil and climatic factors^19^. Consequently, we hypothesized that tree community characteristics and soil characteristics will play indirect roles in driving epiphyte assemblages by providing microhabitat heterogeneity^14^ and nutrients^7^,^8^.

## Results

### Changes in epiphyte abundance and richness along an elevational gradient

**Figure 1:**
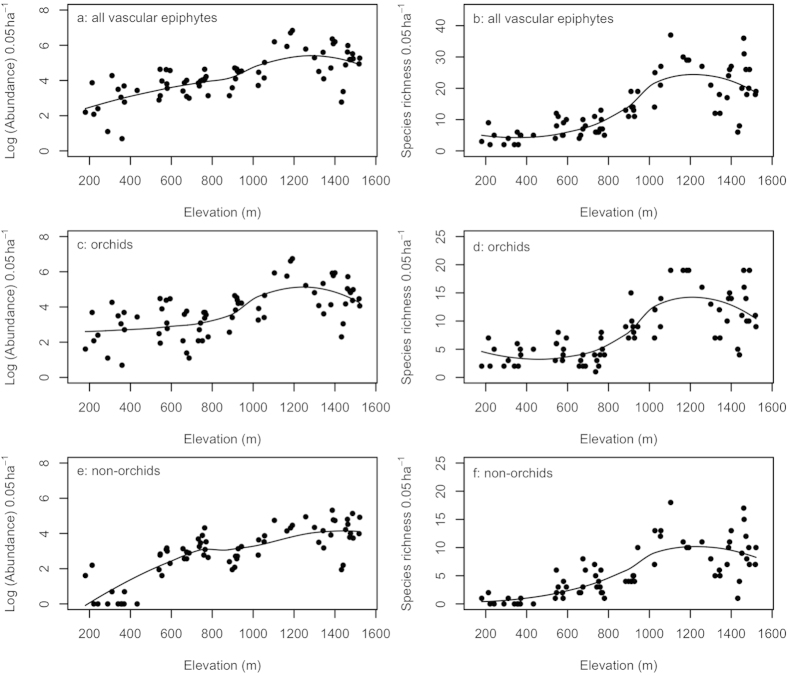
Log of the abundance and number of species for all vascular epiphytes (a,b), orchids (c,d), and non-orchids (e,f) along the elevational gradient. The lines were fitted by using local polynomial regression.

A total of 9272 vascular epiphytes individuals belonging to 111 species, 59 genus and 20 families were recorded. The most abundant species included *Coelogyne fimbriata* Lindley (Orchidaceae), *Pyrrosia lingua* (Thunberg) Farwell (Polypodiaceae), *Liparis bootanensis* Griffith (Orchidaceae), and *Pholidota chinensis* Lindley (Orchidaceae). Orchids contributed 71.3% and 51.4% to the total epiphyte abundance and species richness, respectively. Ferns contributed 22.2% to abundance and 33.3% to species richness. Abundance and species richness of all vascular epiphytes and orchids showed a unimodal relationship with elevation (Fig. 1a–d). Non-orchids showed a unimodal relationship with elevation for species richness, but their abundance followed a linear increase with elevation (Fig. 1e,f). Abundance and species richness increased significantly above 800 m and reached a maximum at 1300 m (Fig. 1). The generalized additive model (GAM) results showed that there were significant positive correlations between elevation and epiphyte abundance (*P* < 0.001) and species richness (*P* < 0.001). Elevation explained more variation in species richness (69.7% and 65.1%) than abundance (57.3% and 49.2%) for all vascular epiphytes and orchids, respectively. For non-orchids, elevation explained more variation in abundance (81.5%) than in species richness (70.8%).

### Changes in epiphyte composition along the elevational gradient

**Figure 2:**
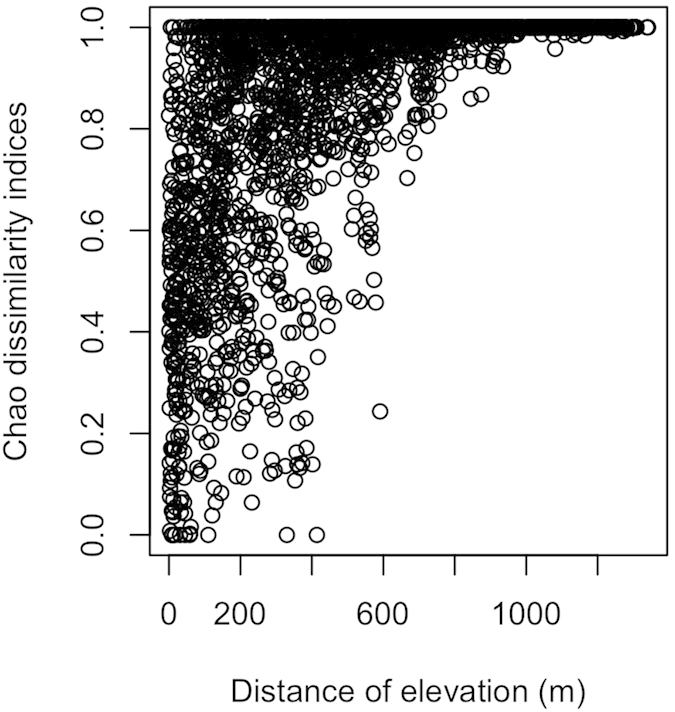
The “chao” dissimilarity indices for the epiphyte communities with the increase of elevation (m).

The non-metric multidimensional scaling (NMS) showed that epiphyte species composition changes along the elevational gradient. The site score of NMS axis 1 of all vascular epiphytes (*R*^*2*^ = 0.833, *P* < 0.001), orchids (*R*^*2*^ = 0.811, *P* < 0.001), and non-orchids (*R*^*2*^ = 0.876, *P* < 0.001) increased significantly with elevation. The species composition of high elevation transects were more similar to each other in NMS space (supplementary Fig. S1). There were no significant spatial correlations between transects and species composition (Mantel test, *R* = −0.066, *P* = 0.85). The dissimilarity indices among transects increased with difference in elevation and also showed high species turnover over relatively short elevation gains (Fig. 2). The results of permutational multivariate analysis of variance (ANOSIM) showed that there were also significantly different variations in species composition of all vascular epiphytes (*F* = 18.125, *P* < 0.001), orchids (*F* = 17.613, *P* < 0.001), and non-orchids (*F* = 18.959, *P* < 0.001) among the seven elevational classes (<400 m, 401–600 m, 601–800 m, 801–1000 m, 1001–1200 m, 1201–1400 m, >1400 m). The indicator species analysis (ISA) was used to identify which epiphytes species occurred at a particular elevational class. The ISA results demonstrated that there were 63 species (56.8%) that showed significant (*P* < 0.05) affinity to one of the seven elevational ranges. There were 21 and 12 indicator species distributed in 1001–1200 m and 1201–1400 m, respectively (Table 1).

### Epiphyte distribution and the determinants along the elevational gradient

**Figure 3:**
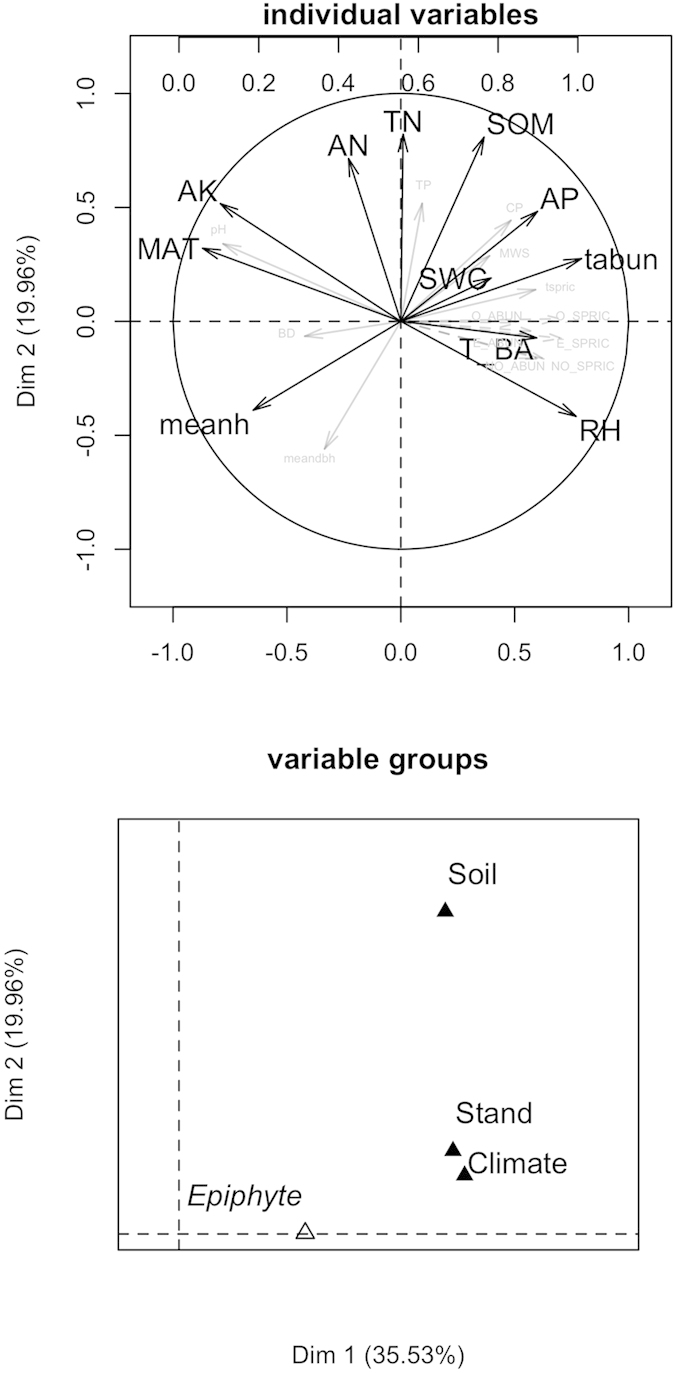
The ordination of different variables using multiple factor analysis (MFA) per transect (0.05 ha) along the elevational gradient. The variables that used in *priori* structural equation model (SEM) are marked with dark arrows. TN, total nitrogen; AN, available nitrogen; AP, available phosphorus; AK, available potassium; SOM, soil organic matter; SWC, soil water content; T_BA, tree basal area; tabun, trees abundance; meanh, mean height of trees; MAT, mean annual temperature; RH, relative humidity.

The first two multiple factor analysis (MFA) dimensions explained 55.5% of the total variation (Fig. 3). The abundance and number of species of all vascular epiphytes, orchids, and non-orchids were significantly (all *P* < 0.001) correlated with the first MFA dimension (Fig. 3) represented by relative humidity, mean annual temperature, community characteristics (tree abundance and basal area) and terrestrial soil physical features as well as aspects of soil fertility (available phosphorus and available potassium). Variables of soil fertility and mean dbh (diameter at breast height) of trees contributed to the second MFA dimension. We used *RV* coefficient (measure of similarity between squared symmetric matrices) to show the closeness of different variable groups in a multivariate matrix. Among the four groups of variables (Fig. 3), climate explained the most variation (*RV* coefficients = 0.341, *P* < 0.001) followed by tree community characteristics (*RV* coefficients = 0.200, *P* < 0.001) and soil features (*RV* coefficients = 0.128, *P* < 0.001).

**Figure 4:**
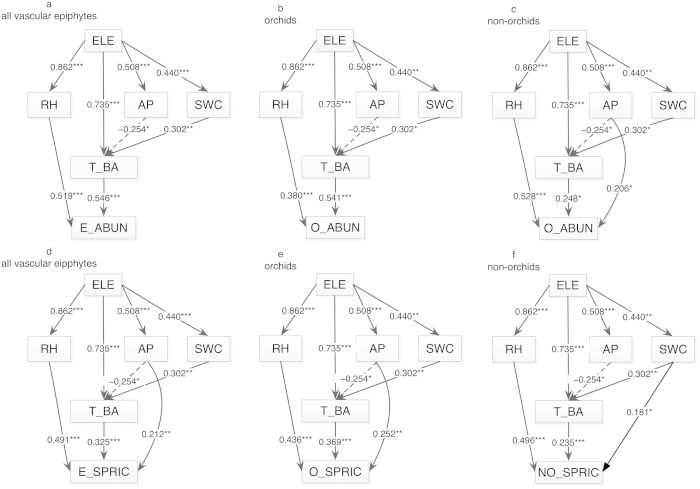
The final structural equation model (SEM) for all vascular epiphytes (a,d), orchids (b,e), and non-orchid (c,f) per transect (0.05 ha) along the elevational gradient. *P < 0.05; **P < 0.01; ***P < 0.001. ELE, elevation; RH, relative humidity; AP; available phosphorus; SWC, soil water content; T_BA, tree basal area; E_ABUN, epiphyte abundance; O_ABUN, orchid abundance; NO_ABUN, non-orchid abundance; E_SPRIC, vascular epiphyte species richness; O_SPRIC, orchid species richness; NO_SPRIC, non-orchid species richness.

A structural equation model (SEM) was used to determine relative importance of direct and indirect effects of abiotic and biotic factors on epiphytes species diversity. The SEMs indicated that the abundance and species richness of all vascular epiphytes or orchids were determined directly or indirectly by different biotic (tree community characteristics) and abiotic (climate and soil) factors (Fig. 4). Generally, relative humidity and tree basal area had the strongest direct effects (positive) on species diversity. Relative humidity and tree basal area had a similar direct effect on all vascular epiphyte abundance and were also significantly indirectly correlated with elevation (Fig. 4a). Orchid abundance showed a similar pattern but tree basal area had a more direct effect. The effect of soil features (available phosphorus and soil water content) on the abundance of all vascular epiphytes or orchids was indirect through tree basal area. The available phosphorus had both indirect and direct effects on the non-orchid abundance. Relative humidity had a more positive effect on the species richness of all vascular epiphytes, orchids, and non-orchids relative to tree basal area (Fig. 4). There were direct significant effects of soil fertility (available phosphorus) but no effect of soil water content on species richness of all vascular epiphyte as well as orchids. Only soil water content had a direct effect on non-orchids richness (Fig. 4f).

## Discussion

### Epiphyte diversity pattern along the elevational gradient

Many studies on vascular epiphytes confirm that montane habitats are more suitable than lowland habitats^3^,^5^,^9^,^11^,^13^,^17^. Rainfall and relative humidity play a significant role in epiphyte establishment and survival^4^. Our study confirms that montane forests host a high diversity of epiphytes, especially in forests categorized as cloud forests^20^. Across our study area, precipitation increases and temperature decreases with elevation. Montane areas in the tropics usually receive additional horizontal precipitation from fog^9^,^21^, which ameliorates the dry season^22^. Many studies present contrasting results on the drivers of epiphyte distribution and diversity. Wolf and Alejandro^10^ confirmed a high diversity belt at mid-elevations (500–2000 m) in neotropical mountains in Chiapas, southern Mexico. In Ecuador, Köster, *et al.*^23^ found there was no significant difference in species richness in lowland rain forests and cloud forests, but their results were affected by an additional water supply in the lowland area.

Our results also show a “mid-elevation bulge” of epiphyte diversity first proposed by Gentry and Dodson^5^ and also found by Cardelus, *et al.*^17^ and Wolf and Alejandro^10^. Species diversity of epiphytes showed unimodal patterns along the elevational gradient. The mid-domain effect arises from geometric constraints on species ranges within abounded domain^24^. However, due to the relative short elevational gradient in our study site, this mid-elevation hump was not as typical as in other sites since the hump in our site is close to the top, not in the middle elevation. And Rahbek^25^ suggested that the lack of a typical mid-elevation hump may be caused by a failure to study a theoretical possible complete gradient in a region. Transects at higher elevations were located in exposed areas with small trees resulting in less space for epiphytes to occupy and strong winds inhibiting establishment. This might explain the decrease in epiphyte abundance and diversity at higher elevations. Conversely, transects at 1200 m belong to tropical montane rain forests or tropical montane evergreen forests, which have more complex community structure and abundant large host trees^26^ for epiphytes to establish.

### Epiphyte compositional variation along the elevational gradient

Our study confirmed high species turnover rate among different transects indicating an obvious environmental gradient and different underlying drivers of community assembly^27^. Species compositional change is further supported by the results of the indicator species analysis. A total of 56.8 % vascular epiphytes species showed affinity to one of the seven elevational ranges. The high percentage of indicator species and great variation in species composition highlights the importance of protecting epiphytes at all elevations, especially for montane areas (1000 m–1400 m). This is treated as an approximation of “fragmentation and narrowness” proposed by Kessler^28^ resulting in geographic isolation and greater endemism^5^,^9^,^18^. Some studies confirm that orchids strongly influence species diversity patterns along environmental gradients^9^. Orchids showed a similar diversity pattern along the elevational gradient as all vascular epiphytes in the study area. Other non-orchids epiphytes (such as ferns) have been shown to facilitate the establishment of orchids^7^,^29^. Consequently, non-orchids also showed the similar patterns to orchids within the elevational gradient of this study.

### Relative effects of abiotic and biotic factors on elevational patterns of epiphyte diversity

In the present study, we found differing importance of abiotic and biotic factors in determining epiphyte abundance. This result is supported by other studies that show the relative importance of determinants on species diversity varies greatly at the community scale^3^,^9^. Elevation indirectly determines epiphyte distributions through its influences on the abiotic and biotic environmental factors such as precipitation, temperature, relative humidity and tree community features^6^,^30^ etc., which directly determine epiphyte distributions. In our study, the indirect effects of elevation influenced epiphyte species richness more than epiphyte abundance. Our results show the dominant role of climate in determining epiphyte diversity. High, stable relative humidity at mid and high elevations provides constant available water for epiphyte growth^5^,^17^,^29^.

Tree community characteristics could play an important role in determining epiphyte diversity. The direct effect of tree basal area on the epiphyte abundance is likely the result of increasing vascular epiphyte diversity with tree size, which could result from an increase in area, time for colonization or microhabitat heterogeneity in canopy crowns^14^. Many studies show that large host trees strongly affect epiphyte abundance and diversity^9^,^14^. We found that basal area of host trees explained a large percentage of the variation in epiphyte species richness. Some physicochemical features (bark characteristics, pH value) of host trees can influence the diversity of epiphytes, but few studies have found strong correlations^31^. For example Boelter, *et al.*^8^ found most epiphyte species demonstrate no significant preference for bark texture. A study from Central American forests has also shown no significant relationship between epiphyte composition and host tree diversity^12^.

The relationship between epiphyte diversity and terrestrial soil nutrients is very complex^8^. Evidence shows that well-developed epiphyte communities can improve nutrient trapping capacity in forest ecosystems^4^,^32^. This demonstrates that epiphytes have a positive effect on the terrestrial soil nutrient accumulation in forests. The effects of soil characteristics on epiphytes are indirect; in Hawaii^7^, soil characteristics have been found to influence trees in the community which in turn influences epiphytes. However, most epiphytes in that experiment were mosses and lichens and there was little information about vascular epiphyte response to the increasing soil nutrients^7^. In our study, soil features seldom had direct effects on epiphytes compared with climate and tree community characteristics. For the epiphytes exclusively inhabiting trees, nutrients and water are taken from the air, rainfall^4^, leaching of leaves and bark, or by decomposing host tree leaves in pockets of canopy soil^7^,^32^. For the vascular epiphytes, edaphic factors had minor effects, especially for moist tropical forests where high nutrient leaching as well as nutrient limitation exists.

Overall, our results showed that different variables have different roles in epiphytes species diversity along the elevational gradient. Among three variables groups, climate variables always played a dominant role in determining species richness and species composition of vascular epiphytes. Climate and tree community features had the strongest direct effects on epiphyte diversity while terrestrial soil variables mainly had indirect effects. Climate related variables explained the most variation in species diversity the elevational gradient. This result demonstrates that the spatial distribution of vascular epiphytes species diversity is more easily predicted at large spatial scales because of the scale of climate data. Due to the significant role of vascular epiphytes in contributing to local species diversity^4^, more work should be done to better understand epiphytes in tropical ecosystems to maintain their biodiversity in the future.

## Methods

### Study site

This study was conducted in the Bawangling Forest Region (BFR; 18°52′–19°12′ N, 108°53′– 109°20′ E) on Hainan Island, south China^21^. This region is the one of largest areas of tropical rain forests in China. The BFR is ca. 500 km^2^ with an elevation range of ca. 100–1654 m a.s.l. The mean annual precipitation varies greatly by elevation, with 1751 mm and 2806 mm at 100 m and 1000 m a.s.l., respectively. The precipitation is seasonally distributed, with a wet season (precipitation ≥ 100 mm/month) from May to October and a dry season (precipitation < 100 mm/month) from November to April^21^. Sixty percent of the area of BFR is protected by Hainan Bawangling National Natural Reserve providing critical habit for Hainan black-crested gibbon (*Nomascus hainanus*), a primate which is listed as critically endangered species by IUCN Red List^33^.

There are six distinct forest types in BFR along the elevational gradient, including tropical deciduous monsoon forest (TDMF, from sea level m 500 m a.s.l), tropical lowland rain forest (TLRF, 0–700 m a.s.l), tropical conifers forest (TCF, 500–700 m a.s.l), tropical montane rain forest (TMRF, 800–1300 m a.s.l), tropical montane evergreen forest (TMEF, 1200–1400 m a.s.l) and tropical dwarf forest (TDF, above 1300 m a.s.l). The TDMF occurs at lower elevations and most canopy species lose their leaves during the dry season. The dominant species are *Terminalia nigrovenulosa* Pierre and *Lagerstroemia balansae* Koehne with an average canopy height of 18 m. The TLRF dominates low elevations around the island but have disappeared rapidly during the last century due to logging. *Cyclobalanopsis patelliformis* (Chun) Y.C. Hsu et H.W. Jen, *Ficus altissima* Blume, *Vatica mangachapoi* Blanco dominate in the canopy and they can reach ca. 28 m in height. The forest canopy of TCF is dominated mainly by one species, *Pinus latteri* Mason, and heights can reach 30 m. Under the canopy, many lowland secondary forest species occur, including *Lithocarpus corneus* (Lour.) Rehder, *Diospyros strigosa* Hemsl. The TMRF is the largest remaining tropical old growth forest on Hainan Island and *Dacrydium pectinatum* de Laubenfels, *Xanthophyllum hainanense* Hu, and *Cyclobalanopsis blakei* (Skan) Schottky dominate in this forest^21^. The TMEF and TDF are considered cloudy forests^20^. The TMEF is adjacent to TDF and is mainly distributed between 1200 m and 1400 m. Dominant species include *Syzygium araiocladum* Merrill & L. M. Perry, *Exbucklandia tonkinensis* (Lecomte) H. T. Chang, *Schima superba* Gardner et Champ with an average canopy height of 18 m. The TDF is mainly distributed around the mountaintops at elevation over 1300 m. *Distylium racemosum* Siebold & Zuccarini dominate in this forest type and canopy only reaches 5–8 m in height. Compared with tropical rain forests in Neotrpical regions and Southeast Asia, the canopy height is relatively low (20–25 m) in our study area creating an easier environment to record vascular epiphytes.

### Data collection

**Figure 5:**
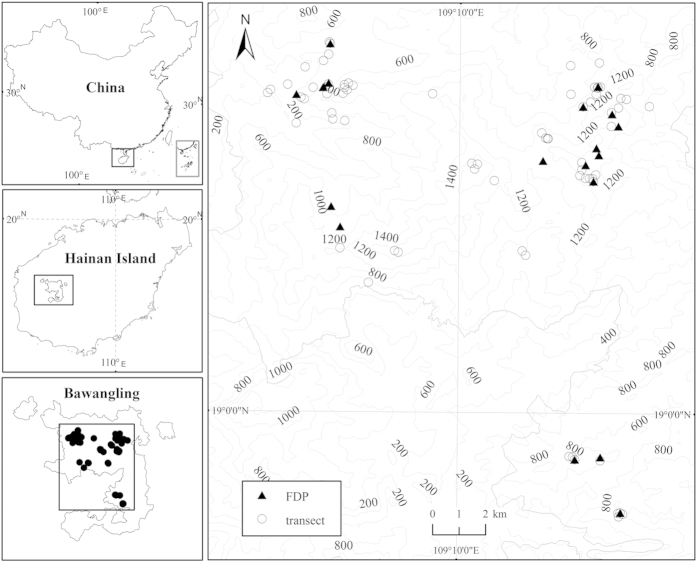
Distribution of the 70 transects inventoried in Bawangling Forest Region by using ArcGIS 9.3 (ESRI, Redlands, CA, USA; http://www.esri.com).

We established 70 transects (10 m × 50 m) to sample vascular epiphyte and tree data along an elevational gradient from 180 m to 1521 m (Fig. 5). Transects were established in old-growth forest communities with minor human disturbance. All transects were placed to avoid river edges and large gaps. The minimum horizontal distance between any two transects was greater than 100 m. Each transect was divided into five 10 m × 10 m quadrats. The quadrats provide a more convenient design to conduct field investigation. We recorded and measured tree species and dbh (diameter at breast height) of all trees ≥10 cm dbh in each quadrat of each transect. Only the trees that were rooted within the quadrant were included. The height of each tree was measured in the field by using altimeter (CGQ-1, Harbin Optical Instrument Factory Co. Ltd, China). The dbh was measured at 50 cm above the end of buttress for the trees with a buttress root. We followed Sandord’s definition of an “individual”: a group of rhizomes and leaves belonging to one species, which forms a clearly delimited stand^34^. Vascular epiphyte species name and number of individuals was recorded for each tree in the field using binoculars, sample pole and single rope climbing^35^. Most epiphytes could be identified and counted from the ground because canopies of most trees were easily visible. When binoculars were insufficient for proper identification, 134 trees (4.1 %) were climbed. Most climbed trees were large trees (≥70 cm dbh) and mainly occurred in the montane rain forest. Only true vascular epiphytes (plants which start and complete their life cycle on the host tree) were included in our study. The field investigations were conducted from March to August and November to December 2008. All species were identified in the field with the help of local botanists. Voucher specimens of unidentified species were sent to South China Botanical Garden of Chinese Academy of Science for final identification. Species’ names of trees and epiphytes follow the *Flora of China* (English edition: http://www.efloras.org/).

Terrestrial soil samples (0–20 cm) were collected in each quadrat and five samples from each transect were mixed to obtain a sample per transect for analysis. All soil samples were air-dried after field collection and transported to a soil laboratory at the Southwest University in Chongqing, China for analysis. Analyses included pH, soil organic matter (SOM), total nitrogen (TN), total phosphorus (TP), available nitrogen (AN), available phosphorus (AP), and available potassium (AK). The detail analysis process and method was given by Long, *et al.*^36^. In each transect, one soil core sample (100 cm^2^) was collected by using cutting ring to determinate the physical properties, including soil water content (SWC), soil bulk density (BD), maximum water holding capacity (MWC), and capillary porosity (CP).

We used climate data from existing Forest Dynamic Plots (FDP) located in the same study area. Total 33 1-ha FDPs that were established according to the method of Center for Tropical Forest Science (CTFS)^37^ to monitor the growth, mortality, and recruitment of all woody species ≥1 cm dbh since 2009. A HOBO Pro Temp/RH data logger (U23-001; Onset, USA) is installed in each FDP to measure the mean annual temperature (MAT, °C) and relative humidity (RH, %). We selected data form 18 old growth FDPs (Fig. 5 and supplementary Table S1) to interpolate climatic variables (MAT and RH) of each transects using a Kriging method with the help of Arc GIS 9.1. For all transects located in the old growth forests, only FDPs belonging to old growth forests were selected to reduce the deviation between old growth forests and recovery forests after disturbance. Among our 70 transects, 12 transects were distributed in 12 1-ha old growth FDPs.

### Data analysis

All analyses were based on aggregated quadrat data from each transect. First, we examined the variation in abundance (E_ABUN) and species richness (E_SPNUM) of vascular epiphytes along elevational gradients using a generalized additive model (GAM). We performed a non-metric multidimensional scaling (NMS) analysis based on ‘‘chao’’ dissimilarity values^38^. The species composition change along the elevational gradient was explored using linear regression. In order to improve visual effects in NMS, transects along the elevational gradient were divided into seven elevation intervals: "<400 m", "401–600 m", "601–800 m", "801–1000 m", "1001–1200 m", "1201–1400 m", ">1400 m". In order to determine the difference in epiphytes species composition along the elevational gradient a permutational multivariate analysis of variance (ANOSIM) was used. The NMS and ANOSIM were used in ‘‘vegan’’ package^39^ in R program^40^. The Mantel test was used to determine the spatial correlation of transects in species composition using “mantel.rtest” in the “ade4” package^41^ in R program^40^. The abundance data was log-transformed before analysis to improve the normality and equality of variance. The “chao” dissimilarity indices were used to explore the change in community composition with the increasing of distance of elevation.

The indicator species analysis (ISA)^42^ was used to determine epiphyte species’ affinity to a specific elevation interval. ISA combines information on a species mean abundance and its frequencies of occurrence in the section (e.g. different elevation intervals in this study). The indicator value (IV) of species is calculated as the product of the relative frequency and relative average abundance in the specific elevation interval. The IV ranges from 0 (no indication) to 1 (perfect indication). The IV reach maximum (IV = 1) when all individuals of a species are found in an elevation interval. The statistical significance of the species indicator values is assessed using a randomization procedure^42^. The species is identified as an significant indicator species when *P* < 0.05. The ISA was conducted by using “labdsv” package^43^ in R program^40^. In this package, *a posteriori* statistical significance of the indicator values is assessed by means of a "Monte Carlo" permutation test^44^.

Second, a multiple factor analysis (MFA) was used to explore the relative importance of different environmental factors that affect species diversity. The MFA was similar to PCA but this method can treat variables of different groups with the same mathematical type^45^. We infer the relative importance of each group of variables instead of single variables by using the *RV* coefficient, which is as a measure of similarity between squared symmetric matrices. In statistics, the *RV* coefficient is a multivariate generalization of the squared Pearson correlation coefficient (because the *RV* coefficient takes values between 0 and 1). It measures the closeness of two sets of points that may each be represented in a matrix. The MFA was conducted in “FactoMineR” package^46^ in R program^40^.

**Figure 6:**
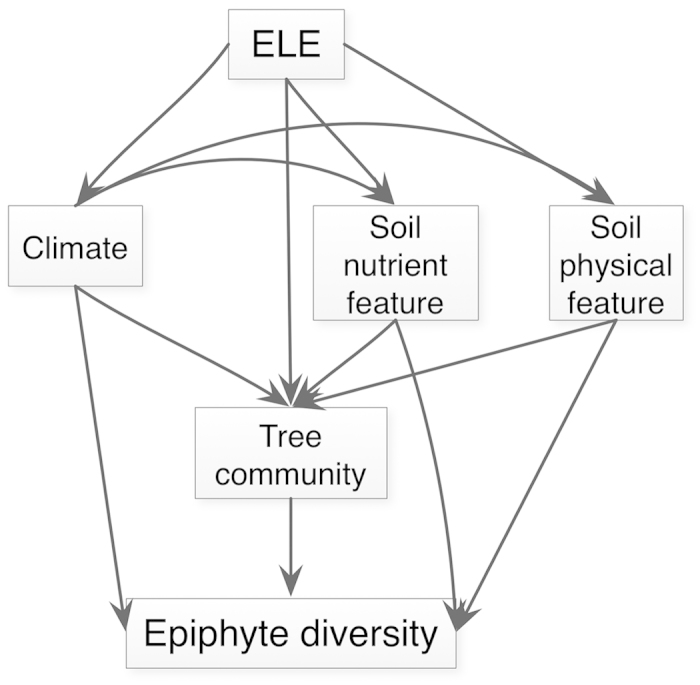
The *a priori* structural equation model (SEM) to distinguish the direct and indirect effect of variables on epiphyte diversity along the elevational gradient.

Third, we used the structural equation model (SEM) to determine the direct and indirect effects of abiotic and biotic factors on epiphytes diversity variables, abundance (E_ABUN) and species richness (E_RICH)^2^,^3^. The abiotic factors included climatic factors (RH, MAT) and edaphic factors (pH, SOM, TN, TP, TK, AN, AP, AK, SWC, BD, MWS, CP). Tree community characteristics included tree stem density (tabun), tree species richness (tspric), tree basal area (T_BA), mean tree dbh (meandbh) and mean tree height (meanh). Before conducting the SEM, the relationship of epiphytes diversity variables (abundance and species richness) with abiotic factors and community characters were examined by using Pearson correlation tests. We removed those dependent variables that showed no significant (*P* < 0.05) relationship with response variables as well as those with low correlation coefficients (*R*^*2*^ < 0.2) in decreasing number of variables used in SEM. After this process, we established an *a priori* SEM to examine the importance of multiple abiotic variables on two diversity indices (abundance and species richness) of epiphytes (Fig. 6). All final SEMs had a *P*-value >0.10 using a chi-square test, 90% confidence intervals of root-mean-square error of approximation (RMSEA) equal to 0, and comparative fit indexes (CFIs) >0.95. We used the “lavaan” package^47^ to conduct the SEM in R program^40^.

## Additional Information

**How to cite this article**: Ding, Y. *et al.* Distribution of vascular epiphytes along a tropical elevational gradient: disentangling abiotic and biotic determinants. *Sci. Rep.* **6**, 19706; doi: 10.1038/srep19706 (2016).

## Supplementary Material

Supplementary Information

## Figures and Tables

**Table 1:** The indicator values of epiphyte species along the tropical elevational gradient.

Species	Family	Elevation (m)	Indicator value	*P* value
*Luisia morsei*	Orchidaceae	<400 m	0.3106	0.005
*Malleola dentifera*	Orchidaceae	<400 m	0.2785	0.007
*Renanthera coccinea*	Orchidaceae	<400 m	0.3496	0.001
*Rhynchostylis gigantea*	Orchidaceae	<400 m	0.4104	0.002
*Robiquetia spatulata*	Orchidaceae	<400 m	0.2727	0.01
*Cleisostoma sp*	Orchidaceae	401–600 m	0.3333	0.004
*Dendrobium spatella*	Orchidaceae	401–600 m	0.5896	0.001
*Pothos repens*	Araceae	401–600 m	0.4133	0.001
*Vanda subconcolor*	Orchidaceae	401–600 m	0.2424	0.019
*Dischidia chinensis*	Asclepiadaceae	601–800 m	0.7416	0.001
*Drymoglossum piloselloides*	Polypodiaceae	601–800 m	0.387	0.001
*Eria rosea*	Orchidaceae	601–800 m	0.3571	0.002
*Hoya pottsii*	Asclepiadaceae	601–800 m	0.4286	0.001
*Phlegmariurus austrosinicus*	Huperziaceae	601–800 m	0.2857	0.011
*Cleisostoma paniculatum*	Orchidaceae	801–1000 m	0.5686	0.001
*Dendrobium aduncum*	Orchidaceae	801–1000 m	0.5	0.001
*Dendrobium changjiangense*	Orchidaceae	801–1000 m	0.6879	0.001
*Dendrobium hainanense*	Orchidaceae	801–1000 m	0.3981	0.001
*Neottopteris nidus*	Aspleniaceae	801–1000 m	0.2213	0.03
*Schefflera arboricola*	Araliaceae	801–1000 m	0.5	0.001
*Scindapsus maclurei*	Araceae	801–1000 m	0.269	0.009
*Asplenium hainanense*	Aspleniaceae	1001–1200 m	0.2302	0.031
*Asplenium neolaserpitiifolium*	Aspleniaceae	1001–1200 m	0.4473	0.002
*Bulbophyllum ambrosia*	Orchidaceae	1001–1200 m	0.4113	0.001
*Bulbophyllum elatum*	Orchidaceae	1001–1200 m	0.3919	0.001
*Bulbophyllum reptans*	Orchidaceae	1001–1200 m	0.25	0.023
*Bulbophyllum retusiusculum*	Orchidaceae	1001–1200 m	0.4253	0.001
*Ceratostylis subulata*	Orchidaceae	1001–1200 m	0.3506	0.005
*Cleisostoma birmanicum*	Orchidaceae	1001–1200 m	0.2959	0.008
*Cymbidium dayanum*	Orchidaceae	1001–1200 m	0.2042	0.047
*Dendrobium williamsonii*	Orchidaceae	1001–1200 m	0.4783	0.001
*Eria pannea*	Orchidaceae	1001–1200 m	0.3464	0.001
*Grammitis adspersa*	Grammitidaceae	1001–1200 m	0.375	0.002
*Grammitis lasiosora*	Grammitidaceae	1001–1200 m	0.321	0.007
*Liparis bootanensis*	Orchidaceae	1001–1200 m	0.2295	0.024
*Pholidota chinensis*	Orchidaceae	1001–1200 m	0.4885	0.001
*Pholidota yunnanensis*	Orchidaceae	1001–1200 m	0.2472	0.014
*Phymatosorus scolopendria*	Polypodiaceae	1001–1200 m	0.5047	0.001
*Prosaptia khasyana*	Grammitidaceae	1001–1200 m	0.5	0.001
*Pseudodrynaria coronans*	Drynariaceae	1001–1200 m	0.3757	0.001
*Pyrrosia tonkinensis*	Polypodiaceae	1001–1200 m	0.25	0.024
*Schoenorchis gemmata*	Orchidaceae	1001–1200 m	0.3861	0.002
*Bulbophyllum kwangtungense*	Orchidaceae	1201–1400 m	0.4572	0.001
*Coelogyne fimbriata*	Orchidaceae	1201–1400 m	0.365	0.001
*Dendrobium sinense*	Orchidaceae	1201–1400 m	0.4373	0.001
*Eria obvia*	Orchidaceae	1201–1400 m	0.2809	0.003
*Eria thao*	Orchidaceae	1201–1400 m	0.3494	0.004
*Hoya lasiogynostegia*	Asclepiadaceae	1201–1400 m	0.2333	0.028
*Humata repens*	Davalliaceae	1201–1400 m	0.4192	0.001
*Lepisorus affinis*	Polypodiaceae	1201–1400 m	0.5556	0.002
*Liparis delicatula*	Orchidaceae	1201–1400 m	0.3702	0.004
*Pyrrosia eberhardtii*	Polypodiaceae	1201–1400 m	0.4673	0.001
*Pyrrosia lingua*	Polypodiaceae	1201–1400 m	0.3703	0.002
*Agrostophyllum callosum*	Orchidaceae	>1400 m	0.4545	0.002
*Eria quinquelamellosa*	Orchidaceae	>1400 m	0.5455	0.001
*Haplopteris flexuosa*	Vittariaceae	>1400 m	0.3135	0.007
*Lepidogrammitis rostrata*	Polypodiaceae	>1400 m	0.355	0.002
*Lepisorus obscurevenulosus*	Polypodiaceae	>1400 m	0.5506	0.001
*Mecodium polyanthos*	Hymenophyllaceae	>1400 m	0.3045	0.005
*Oberonia variabilis*	Orchidaceae	>1400 m	0.2727	0.007
*Peperomia tetraphylla*	Piperaceae	>1400 m	0.2727	0.008
*Phymatopteris obtusa*	Polypodiaceae	>1400 m	0.4061	0.001
*Epigeneium clemensiae*	Orchidaceae	>1400 m	0.2192	0.035
