# Supplementary material for: Distribution of vascular epiphytes along a tropical elevational gradient: disentangling abiotic and biotic determinants
Source: Sci Rep. 2016 Jan 22;6:19706. doi: 10.1038/srep19706 (PMC4726354; doi:10.1038/srep19706)
Supplement: Supplementary Information [file srep19706-s1.pdf]

Distribution of vascular epiphytes along a tropical elevational gradient: disentangling abiotic and biotic determinants

Yi Ding, Guangfu Liu, Runguo Zang, Jian Zhang, Xinghui Lu, Jihong Huang

supplementary information

Fig. S1. The non-metric multidimensional scaling (NMS) for all vascular epiphytes (a), orchids (b), and non-orchids per transect (0.05 ha) along the elevational gradient.

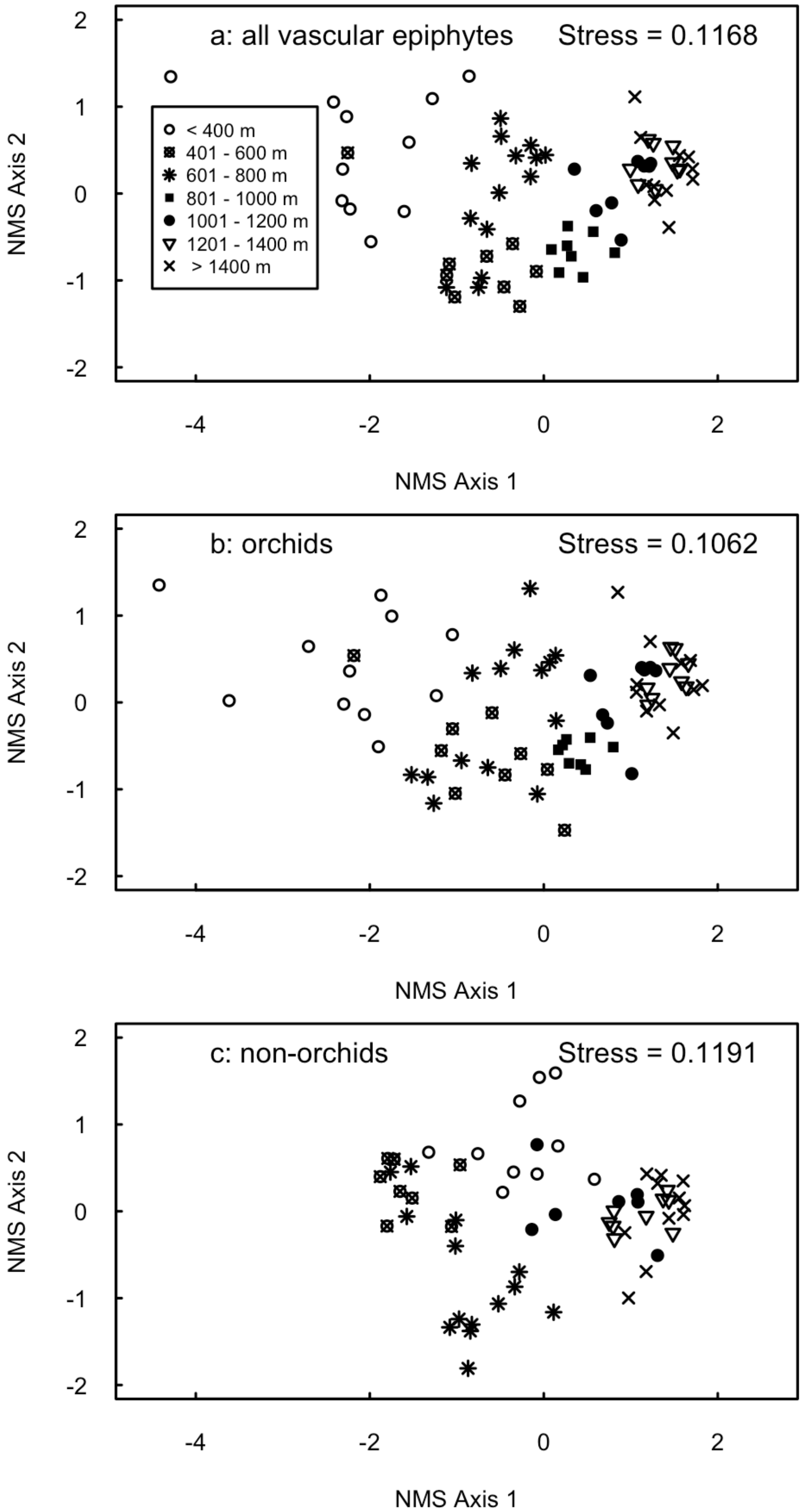

Table S1. The information of 18 FDPs (forest dynamic plot) of tropical forests on Hainan Island.

**FDP**	**Vegetation type**	**Elevation (m)**	**Longitude**	**Latitude**	**Mean annual temperature (℃)**	**Relative humidity (%)**	**Dominate species**
OG12	tropical deciduous monsoon forest	265	109.12088	19.12837	23.25	89.86	*Terminalia nigrovenulosa; Lagerstroemia balansae; Croton laevigatus*
OG11	tropical deciduous monsoon forest	340	109.1091389	19.11058333	22.93	89.02	*Terminalia nigrovenulosa; Streblus ilicifolius; Lagerstroemia balansae*
OG8	tropical lowland rain forest	577	109.1203889	19.11463889	21.81	89.79	*Ficus altissima; Vatica mangachapoi; Cyclobalanopsis patelliformis*
OG2	tropical conifers forest	579	109.2169	18.9848	21.49	91.3	*Pinus latteri;Engelhardtia roxburghiana*
OG10	tropical lowland rain forest	594	109.1184444	19.11311111	21.68	91.2	*Cyclobalanopsis patelliformis ; Vatica mangachapoi*

OG1	tropical conifers forest	666	109.20796	18.98393	21.28	91.46	*Pinus latteri*
OG3	tropical conifers forest	668	109.22406	18.96547	21.29	92.9	*Pinus latteri*
OG9	tropical lowland rain forest	716	109.1216667	19.07166667	20.25	94.28	*Lithocarpus fenzelianus; Vatica mangachapoi*
OG15	tropical montane rain forest	896	109.1248611	19.06458333	19.69	96.37	*Castanopsis hystrix; Castanopsis tonkinensis; Dacrydium pectinatum*
OG16	tropical montane rain forest	923	109.2151944	19.11397222	20.9	90.16	*Castanopsis hystrix; Alseodaphne hainanensis; Xanthophyllum hainanense*
OG17	tropical montane rain forest	1075	109.196	19.088	19.6	94.84	*Castanopsis carlesii; Engelhardia roxburghiana; Castanopsis hystrix; Cryptocarya chinensis; Cyclobalanopsis blakei*
OG18	tropical montane	1084	109.21378	19.08094	19.45	95.79	*Castanopsis carlesii; Castanopsis tonkinensis; Gironniera*

	rain forest						*subaequalis*
OG13	tropical montane evergreen forest	1200	109.2156111	19.09013889	17.85	97.91	*Syzygium araiocladum; Exbucklandia tonkinensis*
OG14	tropical montane evergreen forest	1200	109.2147778	19.09255556	17.79	98.61	*Syzygium araiocladum; Schima superba; Cyclobalanopsis disciformis*
OG7	tropical dwarf forest	1224	109.21	19.107	16.62	97.54	*Distylium racemosum; Syzygium buxifolium*
OG6	tropical dwarf forest	1261	109.2109722	19.08661111	16.49	96.49	*Symplocos poilanei; Rhododendron moulmainense; Distylium racemosum;*
OG5	tropical dwarf forest	1349	109.22242	19.1002	16.56	95	*Distylium racemosum; Syzygium buxifolium*
OG4	tropical dwarf forest	1390	109.2201667	19.10441667	16.39	92.99	*Distylium racemosum; Illicium ternstroemioides*
